# Emerging Functions for N-Terminal Protein Acetylation in Plants

**DOI:** 10.1016/j.tplants.2015.08.008

**Published:** 2015-10

**Authors:** Daniel J. Gibbs

**Affiliations:** 1 School of Biosciences, University of Birmingham, Edgbaston, B15 2TT, UK

**Keywords:** N-terminal acetylation, abscisic acid (ABA), drought stress, plant immunity, protein degradation, N-end rule

## Abstract

N-terminal (Nt-) acetylation is a widespread but poorly understood co-translational protein modification. Two reports now shed light onto the proteome-wide dynamics and protein-specific consequences of Nt-acetylation in relation to plant development, stress-response, and protein stability, identifying this modification as a key regulator of diverse aspects of plant growth and behaviour.

The N-terminus (Nt) of a protein can undergo a wide range of co- and post-translational modifications, including cleavage events, amino acid conjugations, and biochemical alterations [1,2]. Such modifications can have profound effects on protein behaviour, impacting on protein–protein and protein–membrane interactions, subcellular targeting, and protein stability, which is dependent on the N-end rule pathway of proteolysis [1,2]. One of the most widespread Nt-modifications in eukaryotic organisms is Nt-acetylation, where acetyl moieties are transferred from acetyl-CoA to the exposed α-amino group of the Nt-residue [3]. This modification typically occurs co-translationally, and, in contrast to internal lysine acetylation, is irreversible [3]. Nt-acetylation of nascent polypeptides is carried out by N^α^t-acetyltransferases (NATs), which are protein complexes comprising of a catalytic and auxiliary subunit. Three main NATs account for the majority of Nt-acetylation events in yeast and humans by targeting distinct N-termini; NATA acetylates certain exposed amino acids following Nt-Met excision, whereas NATB and NATC target Nt-Met residues preceding acidic or hydrophobic residues, respectively [1,3].

Despite the fact that more than 80% of proteins in humans and plants undergo Nt-acetylation [4], the functional relevance of this modification has remained elusive. A recent significant breakthrough in yeast and mammals was the discovery that Nt-acetylation can target proteins for degradation via a novel branch of the N-end rule pathway [5]. It was postulated that all Nt-acetylated proteins can in principle be targeted by this ‘Ac/N-end rule pathway’, but proteolysis via this mechanism is conditional and dependent on exposure of the N-terminus, which may occur, for example, when a protein is misfolded or protein complexes are disrupted [2,5,6].

There is a lack of studies on Nt-acetylation in plants, although phenotypes associated with NAT loss of function in *Arabidopsis* have been reported. For example, loss of NATC activity negatively affects photosynthetic efficiency [7], whereas mutation of the NATB auxiliary subunit causes a range of pleiotropic defects [8]. It is not known whether any of these phenotypes are due to downstream changes in protein stability, or other putative Nt-acetylation functions as previously described in animal and yeast systems (e.g., subcellular mis-targeting, abolishment of protein interaction or disruption of membrane association [3]). Two new papers by Linster *et al.* [9] and Xu *et al.* [10] have now added to our knowledge of the conservation and importance of Nt-acetylation in plants, particularly in relation to the management of abiotic stress-tolerance, immunity and protein stability. Importantly, these recent findings have general implications for the cross-kingdom functions of this enigmatic chemical modification as an essential and dynamic regulator of protein behaviour and cellular activity.

Linster *et al.* functionally characterised the plant NATA complex [9]. They found that NATA specificity in plants is conserved, and that its function is indispensable, since T-DNA null mutants of either NATA subunit were embryo lethal. A microRNAi approach was therefore used, which led to the production of plants with retarded growth and significant increases in the global levels of non-acetylated N-termini. NATA-depleted plants were highly drought-tolerant, relative to wild type. The extreme drought-tolerance was due to altered root morphology and reduced stomatal aperture, prompting the hypothesis that it might be associated with alterations in signalling of the abiotic stress phytohormone absicisic acid (ABA). Validating this theory, NATA RNAi plants were shown to constitutively express key ABA- and drought-associated genes. Remarkably, it was found that drought stress or exogenous application of ABA to wild type *Arabidopsis* caused a rapid depletion of NATA transcript and protein abundance, concomitant with a reduction in the total number of Nt-acetylated proteins, suggesting that decreases in global Nt-acetylation are a specific and functionally important response triggered by abiotic stress (Figure 1A). This highlights a novel and agronomically relevant significance for this modification in cellular stress-surveillance, and suggests that NATA-regulated modulation of the Nt-acetylome contributes to plant plasticity under drought, and perhaps other ABA-associated stresses. The mechanistic consequence of altered proteome-wide Nt-acetylation is currently unknown. Nonetheless, this study reveals that protein Nt-acetylation is dynamic and signal-responsive, and that global change in the levels of this biochemical modification can have profound effects on plant growth and development.

A second report by Xu *et al.* focussed on the Nt-acetylation dynamics of the Nod like receptor (NLR) protein SUPPRESSOR OF NPR1, CONSTITUTIVE 1 (SNC1), revealing the functional relevance of its modification in relation to plant immunity [10]. NATA was identified as a modulator of SNC1-mediated response to pathogens; SNC1 protein accumulated in NATA mutant plants and enhanced pathogen-tolerance. This suggests that Nt-acetylation of SNC1 might act as a degradation signal. Remarkably, proteomic analyses identified two distinct Nt-variants of SNC1: (i) Nt-Met-Met-Asp-SNC1, which is Nt-acetylated by NATA, and (ii) Nt-Met-Asp-SNC1, which lacks the first Nt-Met residue and is Nt-acetylated by NATB (Figure 1B). These variants are likely generated via alternative initiation. Intriguingly, Nt-acetylation of these alternative N-termini has contrasting effects on protein abundance, destabilising SNC1 when the first Met is present, but stabilising SNC1 when the second Met is Nt-acetylated. Thus, Nt-acetylation can have antagonistic effects on a single protein's half-life, depending on the sequence context of the modification [10]. This implies that the relationship between Nt-acetylation and proteolysis is more complex than previously postulated [5]. It will now be crucial to identify the downstream E3 ligase(s) recognising the Nt-acetylation degron in SNC1, as well as determining whether this differential targeting is signal-responsive and widespread. It is tempting to speculate that other proteins initiating with a double Met might also be subjected to this dual regulation.

It has recently been proposed that Nt-acetylation may be more dynamic than previously considered, playing a regulatory role in growth and development [11]. These new plant studies also support this notion. Furthermore, a plastid-specific NAT has recently been identified [12], and chloroplastic proteins are Nt-acetylated post-translationally, following a transit-peptide cleavage step [4]. This implies that compartmentalisation of NAT-activity is functionally important. There has been growing interest in the study of Nt-modifications in plants in recent years, particularly in relation to the N-end rule pathway of proteolysis [2]. It will now be important to determine whether the recently identified Ac/N-end rule pathway functions in plants. Homologues of the relevant E3 ligases are present in plant genomes [2], and the effects of NAT activity on SNC1 turnover [and a second NLR, RESISTANCE TO *P. syringae pv maculicola* 1 (RPM1)] also support this proposition [10]. Another pertinent question is how ABA- and pathogen-associated stress signals impact on NAT function. Effects on transcription and protein-depletion are implicated by Linster *et al.*, but it is also possible that these signals may modulate NAT enzymatic activity in other ways, for example via post-translational modifications.

It is becoming increasingly apparent that what was previously considered a constitutive and inert modification actually has a great deal of functional significance, at protein-specific and proteome-wide levels. The scene is now set for further studies into the complexity and functional relevance of this widespread but enigmatic modification in plants and beyond.

## Figures and Tables

**Figure 1:**
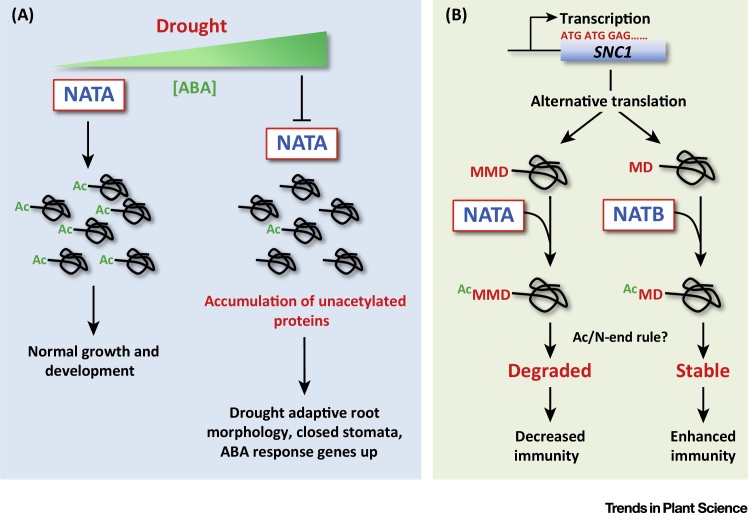
Recently Identified Functions for N-Terminal (Nt) Acetylation in Plants. (A) Under normal conditions NATA co-translationally Nt-acetylates (Ac) a large number of proteins following Nt-Met excision [1,3]. Drought-induced abscisic acid (ABA) accumulation (green gradated triangle) depletes NATA transcript and protein abundance, which leads to a global reduction in the number of Nt-acetylated proteins, resulting in several adaptive responses that improve drought-tolerance and survival [9]. Thus, NATA-mediated Nt-acetylation is proposed to act as an important switch coordinating metabolic, developmental and physiological responses downstream of ABA. (B) The Nod-like receptor protein SNC1 plays a key role in plant immunity. Two Nt-variants of SNC1 are present in plants, most likely arising as a result of alternative translation. Nt-Met-Met-Asp-SNC1 (MMD-) is Nt-acetylated by NATA, whereas Nt-Met-Asp-SNC1 (MD-), which lacks the first Met residue, is Nt-acetylated by NATB [10]. Remarkably, these Nt-acetylation events have contrasting consequences, destabilising or stabilising SNC1, which decreases or enhances the immune response, respectively. Xu *et al.* suggest that Nt-acetylation of these alternate Nt-isoforms contributes to overall SNC1 homeostasis. This study reveals that different NAT complexes can have antagonistic effects on the stability of a protein depending on the nature of its N-terminus, suggesting that: (i) control of protein half-life by Nt-acetylation is more complex than previously postulated [5,6], and; (ii) that the previously identified acetylation-dependent branch of the N-end rule pathway (the Ac/N-end rule) may be functional in plants [2].

## References

[1] Giglione C. (2015). N-terminal protein modifications: Bringing back into play the ribosome. Biochimie.

[2] Gibbs D.J. (2014). The eukaryotic N-end rule pathway: conserved mechanisms and diverse functions. Trends Cell Biol..

[3] Starheim K.K. (2012). Protein N-terminal acetyltransferases: when the start matters. Trends Biochem. Sci..

[4] Bienvenut W.V. (2012). Comparative large scale characterization of plant versus mammal proteins reveals similar and idiosyncratic N-alpha-acetylation features. Mol. Cell. Proteomics.

[5] Hwang C.S. (2010). N-terminal acetylation of cellular proteins creates specific degradation signals. Science.

[6] Shemorry A. (2013). Control of protein quality and stoichiometries by N-terminal acetylation and the N-end rule pathway. Mol. Cell.

[7] Pesaresi P. (2003). Cytoplasmic N-terminal protein acetylation is required for efficient photosynthesis in Arabidopsis. Plant Cell.

[8] Ferrandez-Ayela A. (2013). Mutation of an Arabidopsis NatB N-alpha-terminal acetylation complex component causes pleiotropic developmental defects. PLoS ONE.

[9] Linster E. (2015). Downregulation of N-terminal acetylation triggers ABA-mediated drought responses in Arabidopsis. Nat. Commun..

[10] Xu F. (2015). Two N-terminal acetyltransferases antagonistically regulate the stability of a nod-like receptor in Arabidopsis. Plant Cell.

[11] Silva R.D., Martinho R.G. (2015). Developmental roles of protein N-terminal acetylation. Proteomics.

[12] Dinh T.V. (2015). Molecular identification and functional characterization of the first Nalpha-acetyltransferase in plastids by global acetylome profiling. Proteomics.

